# Glycation reactions of methylglyoxal during digestion in a dynamic, in vitro model of the upper gastrointestinal tract (TIM‐1)

**DOI:** 10.1002/fsn3.4118

**Published:** 2024-03-25

**Authors:** Stephanie Treibmann, Koen Venema, Thomas Henle

**Affiliations:** ^1^ Chair of Food Chemistry Technische Universität Dresden Dresden Germany; ^2^ Centre for Healthy Eating & Food Innovation (HEFI) Maastricht University – Campus Venlo Venlo The Netherlands

**Keywords:** creatine, digestion, glycation, methylglyoxal, TNO in vitro gastrointestinal digestion model

## Abstract

The 1,2‐dicarbonyl compound methylglyoxal (MGO) can react with and thereby impair the function of proteins and DNA, leading to pathophysiological pathways in vivo. However, studies on the bioavailability of dietary MGO and its reactions during digestion have diverging results. Therefore, simulated digestion experiments of MGO, protein, and creatine were performed in the dynamic, in vitro model of the upper gastrointestinal tract (TIM‐1). This multicompartment model continuously adjusts pH values and has realistic gastrointestinal transit times while also removing water and metabolites by dialysis. Samples were analyzed with HPLC‐UV for MGO and HPLC–MS/MS for creatine and glycated amino compounds. MGO reacted with creatine during simulated digestion in TIM‐1 to form the hydroimidazolone MG‐HCr in similar amounts as in a human intervention study. 28%–69% of MGO from the meal were passively absorbed in TIM‐1, depending on the addition of creatine and protein. Simultaneous digestion of MGO with ovalbumin led to the formation of the lysine adduct *N* ^*ε*^‐carboxyethyllysine (CEL) and the methylglyoxal‐derived hydroimidazolone of arginine (MG‐H1). The formation of both compounds decreased with added creatine. Hence, glycation compounds are formed during digestion and significantly contribute to other ingested dietary glycation compounds, whose physiological consequences are critically discussed.

## Graphical abstract

We used TIM‐1, an elaborate model of human gastric and small intestinal digestion, to investigate the bahavior of methylglyoxal (MGO) during digestion and found that up to 69% of MGO remains available for resorption. Our study supports the hypothesis that glycation compounds are formed in the gastrointestinal tract following uptake of dietary dicarbonyl compounds. These “dAGEs” represent a third source of glycation compounds (besides dietary compounds, which are taken up directly with the diet, and endogenously formed compounds).

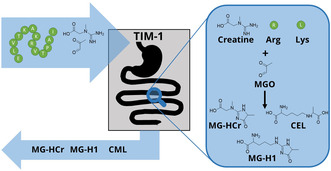

## 1 INTRODUCTION

Methylglyoxal (MGO) is a 1,2‐dicarbonyl compound formed during the heat treatment, storage, and fermentation of carbohydrate‐rich foods. It is highly reactive toward amino compounds like protein and DNA and forms glycated amino acids as well as color and aroma compounds. Concentrations in food range from a few mg/kg MGO in juices, beer, and baked goods to up to 500 mg/kg in Manuka honey (Degen et al., 2012). The daily intake of MGO is estimated to be between 0.04 (Maasen et al., 2021) and 0.3 mmol (Degen et al., 2012). MGO is also formed as a byproduct of glycolysis (approx. 3 mmol/d) and other processes in vivo (Rabbani & Thornalley, 2012). When its formation is increased, for example, during hyperglycemia, and/or the metabolization via the glyoxalase system is decreased, MGO can react with and thereby impair endogenous proteins (Rabbani & Thornalley, 2012). Therefore, methods to lower MGO concentrations in vivo by introducing scavengers or altering glyoxalase activity have been researched (Rabbani et al., 2016).

For some years, the influence of dietary MGO on in vivo levels as well as physiological outcomes has been critically discussed. One study found that MGO intake did not increase concentrations of MGO or its metabolite, d‐lactate, in urine (Degen et al., 2013). However, higher habitual MGO intake correlated with higher plasma MGO concentrations and skin autofluorescence in a recent study (Maasen, Eussen, Scheijen, et al., 2022). The same cohort had less low‐grade inflammation in participants with a higher habitual intake of MGO (Maasen, Eussen, Dagnelie, et al., 2022). Prolonged exposure to high levels of MGO in animals induced various negative effects like decreased locomotive behavior and promoted amyloid β accumulation in *Caenorhabditis elegans* (Wei et al., 2022) and oxidative stress and endothelial dysfunction in rats (Sena et al., 2012). On the other hand, long‐term oral application of MGO had no adverse effects on physical performance and organs in rabbits, rats, mice, and dogs (Ghosh et al., 2006). Additionally, low doses of MGO even increased lifespan in *C. elegans* (Ravichandran et al., 2018).

To assess the physiological consequences of dietary MGO, it is crucial to understand how much MGO is available for absorption, how much is transported to the colon, what reaction products are formed, and how co‐ingestion of proteins and other amino compounds affects all of this. There are many studies on simulated digestion experiments performed at 37°C with a 2 h gastric phase at pH 2 and a 1–6 h intestinal phase at pH 7 or 8 with added enzymes and digestive fluids. Some studies have an additional oral stage for 2 min at 37°C (Hamzalıoğlu & Gökmen, 2016; Jiang et al., 2019). Typically, 10%–50% of MGO are left after 3–8 h simulated digestion, which might explain its poor bioavailability (Amoroso et al., 2013; Daglia, Ferrari, et al., 2013; Degen et al., 2013; Hamzalıoğlu & Gökmen, 2016; Jiang et al., 2019; Treibmann et al., 2022). Simultaneous digestion with amino acids like lysine and cysteine or protein decreased MGO concentrations after digestion, while simulated digestion experiments without enzymes and bile showed only little MGO loss (Hamzalıoğlu & Gökmen, 2016; Jiang et al., 2019; Treibmann et al., 2022). MGO mainly reacts with amino and sulfur compounds like arginine, creatine, and cysteine during digestion to form glycated amino compounds like the MGO‐derived hydroimidazolone of arginine (MG‐H1) and creatine (MG‐HCr) (Treibmann et al., 2022). These reaction products formed during digestion can contribute to dietary glycation compounds whose pathophysiological role is critically discussed (Nowotny et al., 2018).

However, the above‐described simulated digestion experiments are static and therefore drastically differ from the human gastrointestinal tract, which has alternating pH levels, peristaltic movement, continuous lumen transition, and the removal of nutrients by passive and active absorption (Guerra et al., 2012). This difference influences the reaction of methylglyoxal and creatine during digestion. In static simulated digestion experiments, 56% of the initial MGO reacted with creatine to form the hydroimidazolone MG‐HCr. During a human intervention study, where participants consumed MGO from Manuka honey together with creatine, only 0.2% of the ingested MGO was recovered as MG‐HCr from urine. Creatine is rapidly passively absorbed after consumption, which is not implemented in most simulated digestion models (MacNeil et al., 2005). The gastrointestinal model of the stomach and small intestine (TIM‐1) of the Dutch Organization for Applied Scientific Research (TNO) is a dynamic computer‐controlled multicompartmental model of the human upper gastrointestinal digestion. In TIM‐1, pH values are continuously adjusted, and realistic gastric and small intestinal transit times and flow rates are simulated, while also removing water and metabolites by dialysis, simulating bioavailability (Minekus et al., 1995). It accurately predicts the behavior of nutrients during digestion compared to in vivo data (Minekus, 2015).

The goal of this study, therefore, was to perform simulated digestion experiments of MGO with added protein or creatine in TIM‐1 to study the following hypotheses:

1. TIM‐1 is a more suitable model to study the reactions of MGO during digestion than static models without simulated absorption;
2. MGO is unstable during digestion due to reactions with amino acids and amines, which form glycation compounds; and
3. Creatine protects amino acids from derivatization with MGO during digestion.

## 2 MATERIALS AND METHODS

### 2.1 Chemicals

Creatinine and sodium borohydride were obtained from Merck (Darmstadt, Germany), hydrochloric acid from VWR ProLabo (Leuven, Belgium), creatine monohydrate from AppliChem (Darmstadt, Germany), and acetic acid from Roth (Karslruhe, Germany). Sodium hydroxide, albumin from chicken eggs, HPLC gradient grade methanol, and LC–MS grade acetonitrile were purchased from Fisher Chemicals (Loughborough, UK), isotopically labeled ^2^D_3_‐creatine and ^2^D_3_‐creatinine from Cambridge Isotope Laboratories (Loughborough, UK), and formic acid, sodium borate, sodium dihydrogen phosphate, and disodium hydrogen phosphate from Grüssing (Filsum, Germany). Water for solutions, buffers, and HPLC–MS/MS was prepared with a Bi 18 E double distillation system (QCS, Maintal, Germany). Sodium caseinate was isolated, according to Moeckel et al. (2016). MG‐HCr, D_3_‐MG‐HCr (Löbner et al., 2015), *N* ^ε^‐carboxymethyllysine (CML), D_2_‐CML (Hellwig et al., 2011), *N* ^ε^‐carboxyethyllysine (CEL), ^13^C_3_‐CEL (Hellwig et al., 2011), MG‐H1, and ^13^C_6_‐MG‐H1 (Hellwig et al., 2011) were synthesized and characterized in our laboratory according to the literature stated. The substances met the spectroscopic and chromatographic characteristics published in the respective protocols. All other chemicals were procured from Sigma‐Aldrich (Steinheim, Germany).

### 2.2 TNO in vitro model of the stomach and small intestine (TIM‐1)

The in vitro model of the stomach and small intestines was set up as described before for protein digestion under the average physiological conditions found in the human gastrointestinal tract (Minekus et al., 1995; van der Lugt et al., 2020). A schematic diagram of the setup can be found as Supporting Information (Figure S1). Gastric and intestinal pH curves, gastric emptying, and intestinal residence time were modeled after human digestion for semi‐solid foods (Figure S2) (Minekus et al., 1995). Briefly, the model consists of 4 compartments (stomach, duodenum, jejunum, and ileum), each composed of outer glass containers and inner flexible silicone tubes. The space in between glass and silicone is filled with water at 37°C, which is pumped periodically to simulate the peristaltic movement of the silicone tubes. Hollow fiber membranes (DICEA 90G, Baxter, Deerfield, IL, USA) were connected to the jejunum and ileum compartments to dialyze low‐molecular‐weight compounds and simulate absorption of the nutrients. At the start of the experiment, the meal is applied to the gastric compartment. The other compartments are filled with starting residue, as described before (van der Lugt et al., 2020). The meal is continuously transitioned through the model at rates modeled after in vivo studies (Figure S2) (Minekus et al., 1995). The composition of secreted enzymes, electrolytes, bile, and pancreatic juice was adjusted to the average concentrations as described for adults after ingestion of a meal. The pH is computer‐controlled and adjusted by secreting hydrochloric acid (gastric compartment) and bicarbonate (intestinal compartments). Pancreatic output was simulated by secreting 10% pancreatin (amylase 12,000 IU/g, protease 1000 IU/g, and lipase 15,000 IU/g) in a small intestinal electrolyte solution (containing NaCl 5 g/L, KCl 0.6 g/L, and CaCl_2_ 0.22 g/L) at 0.25 mL/min. Biliary output was simulated by secreting a 4% bile (porcine bile extract, Sigma) solution at 0.5 mL/min. The test meals consisted of 150 mL of gastric electrolyte solution (CaCl_2_ 0.22 g/L, KCl 2.2 g/L, NaCl 5 g/L, and NaHCO_3_ 1.5 g/L), 45 mL of 1 M citrate buffer, 5.8 mL of gastric enzyme solution (180,000 IU pepsin and 11,250 IU lipase in 150 mL of gastric electrolyte solution and 1.5 mL of 1 M sodium acetate). The test substances were added (142 μL of 40% MGO, 2.917 g of creatine monohydrate, 17.5 g of ovalbumin, and 19.7 g of sodium caseinate), and the volume was brought to 350 mL with water. The pH of the meal was adjusted to 6.5 with hydrochloric acid, and 300 g of the meal was introduced into the gastric compartment of TIM‐1. Gastric experiments lasted 3 h, and samples were collected hourly at the ‘pyloric sphincter’ (Figure S1, B). For experiments of the complete model, samples were collected hourly for 6 h from the jejunal and ileal dialysate and the ileal efflux (Figure S1, M and H). The residues remaining in the model after termination, as well as samples of the secretion fluids and the meal, were also collected. Samples for MGO analysis (500 μL) were immediately added to 1 mL of methanol, while samples for amino acid, creatine, MG‐HCr, and glycated amino acid analysis (10 mL) were added to 1 mL of amino guanidine (53 mM for efflux and residue samples, 5.3 mM for dialysates) to remove residual MGO. Samples were stored at −18°C until analysis.

### 2.3 Analysis of α‐amino nitrogen (AAN)

For AAN analysis, a method was adapted from the literature (Keller et al., 2017). Shortly, 196 μL of 0.5 M phosphate buffer (pH 8, with 3 mg/mL sodium dodecyl sulfate and 0.1 mg/mL sodium sulfite) was added to 20 μL of sample. After mixing and heating at 40°C for 1 min, 27 μL of the sample was added to 100 μL of 1.35 mM 2,4,6‐trinitrobenzenesulfonic acid. After heating at 40°C for 40 min, absorbance was measured at 405 nm. Glycine was used for external calibration.

### 2.4 Analysis of creatine, amino acids, and glycation compounds

Samples for creatine, creatinine, and MG‐HCr analysis were prepared and subjected to high‐pressure liquid chromatography coupled to tandem mass spectrometry (HPLC‐MS/MS), as described previously (Treibmann et al., 2019, 2020, 2022). For the analysis of protein‐bound MG‐H1, CML, and CEL, published protocols for acidic hydrolysis were adjusted (Hegele et al., 2008; Scheijen et al., 2016), and samples were applied to HPLC‐MS/MS. Amino acids were analyzed on an amino acid analyzer (Treibmann et al., 2017). Methylglyoxal was derivatized with o‐phenylenediamine and subjected to HPLC as published previously (Degen et al., 2012; Rückriemen et al., 2017). Detailed methods are described in the Supporting Information.

### 2.5 Data treatment

All TIM‐1 experiments were conducted in duplicate. The results are presented as means with standard deviations. Standard deviations were calculated as described in Dean and Dixon (1951) for small numbers of observations from the range *r* and the deviation factor *k* (*k* = 0.89 for two observations) as s=k∙r. The concentrations measured in the TIM‐1 fractions were each multiplied by the volume of the fraction to obtain the absolute amount of substance. A control run with water was used to correct for the analytes present in the digestive fluids. These amounts were subtracted from the concentrations in the samples. Chain length of peptides were calculated as the quotient of α‐amino nitrogen and total amino acid nitrogen as resulting from amino acid analysis, thus representing the medium chain length of peptides in the dialyzed fractions.

## 3 RESULTS AND DISCUSSION

### 3.1 TIM‐1 as a model to study the reactions of methylglyoxal during digestion

The aim of this study was to analyze the reactions of MGO during digestion. To do this, we first wanted to assess whether TIM‐1 is a more suitable model to study the reactions of MGO during digestion compared to static simulated digestion experiments. Therefore, we compared simulated digestion experiments of methylglyoxal and creatine in TIM‐1 with digestion experiments of the two substances in static simulated digestion models and in human intervention studies.

TIM‐1 has four compartments, simulating the stomach, duodenum, jejunum, and ileum. Samples are taken from two dialysis units that are attached to the jejunum and the ileum compartment, respectively. These dialyzed fractions mimic passive absorption. The ileum efflux is also collected and represents intestinal content transported to the colon.

**FIGURE 1:**
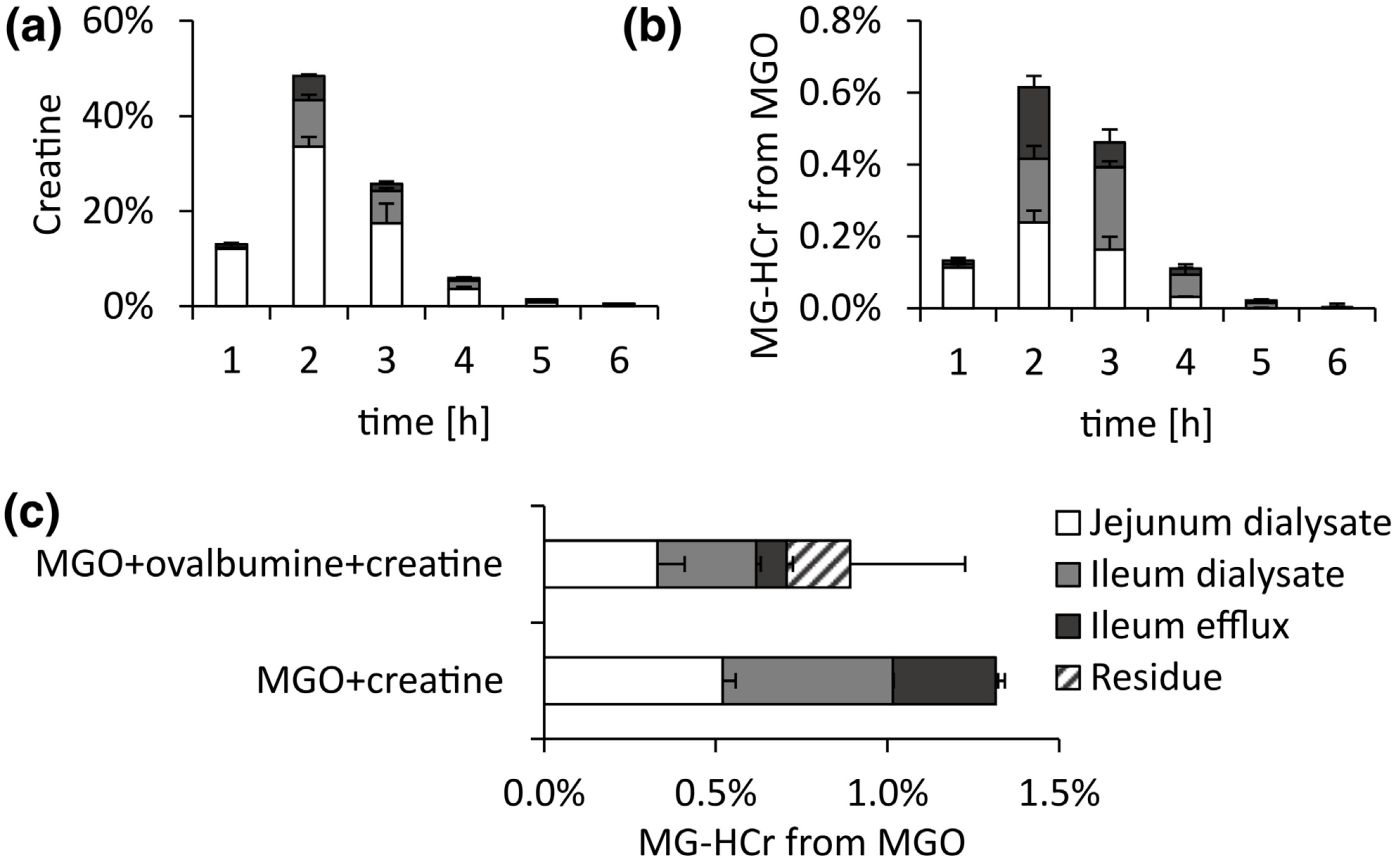
Hourly contents of creatine (a) and newly formed MG‐HCr (b) in the dialysates and efflux of TIM‐1 during simulated digestion experiments of MGO and creatine, and total MG‐HCr formation in simulated digestion experiments of MGO and creatine with and without added ovalbumin (c) using TIM‐1. Data are presented as mean ± SD, *n* = 2.

As shown in Figure 1a and Figure S3a–c, creatine was quickly passively absorbed in TIM‐1 both with and without added MGO and protein, with the highest amount of creatine found in the jejunum dialysate after 2 h. This is in accordance with studies where human participants consumed creatine and the maximum of creatine in plasma was detected after 50–100 min (Rawson et al., 2002; Steenge et al., 2000). The total recovery of creatine in the system was 96 ± 5%, and less than 1% of creatine reacted to form creatinine in all runs with creatine in the meal (Figure S3e,f).

The reaction product of creatine and MGO, MG‐HCr, was formed during simulated digestion using TIM‐1 (Figure 1b). Its amounts were highest after 2 h, and they were found to be equally distributed between jejunum and ileum dialysate and ileum excretion fractions, indicating a delay compared to creatine absorption. This might be due to the reaction kinetics of MG‐HCr formation. Of the initially submitted MGO, 1.3 ± 0.2% reacted with creatine to form MG‐HCr (Figure 1c), and 1.0 ± 0.1% was passively absorbed as MG‐HCr. These recoveries are considerably lower compared to a simulated digestion using a static system without absorption, where 56% of MGO reacted to form MG‐HCr (Treibmann et al., 2022). The reason for this is presumably the quick absorption and thereby removal of creatine and MGO from the system by the dialysis units of TIM‐1, which corresponds more to the actual physiological conditions. With less time to react, less MG‐HCr can be formed. On the other hand, the MG‐HCr absorption in TIM‐1 experiments is similar to the formation of MG‐HCr in a human intervention study. In this study, 0.3% of MGO consumed simultaneously with creatine was excreted as MG‐HCr (Treibmann et al., 2022). Since creatine is quickly absorbed in humans, a similar explanation for lower recoveries applies. Therefore, TIM‐1 is a more suitable model to study the reactions of MGO during digestion compared to static digestion models without dialysis or absorption.

However, the main limitation of TIM‐1 is the absence of epithelial cells. The dialysis units of TIM‐1 only model passive absorption; no active uptake, reactions with cell components, or metabolization can be considered. In addition, TIM‐1 also has no mucus layer, which mainly consists of glycosylated proteins. These layers could also react with or retain MGO. These influences could be studied in additional cell culture experiments.

### 3.2 Digestion of methylglyoxal without additives

**FIGURE 2:**
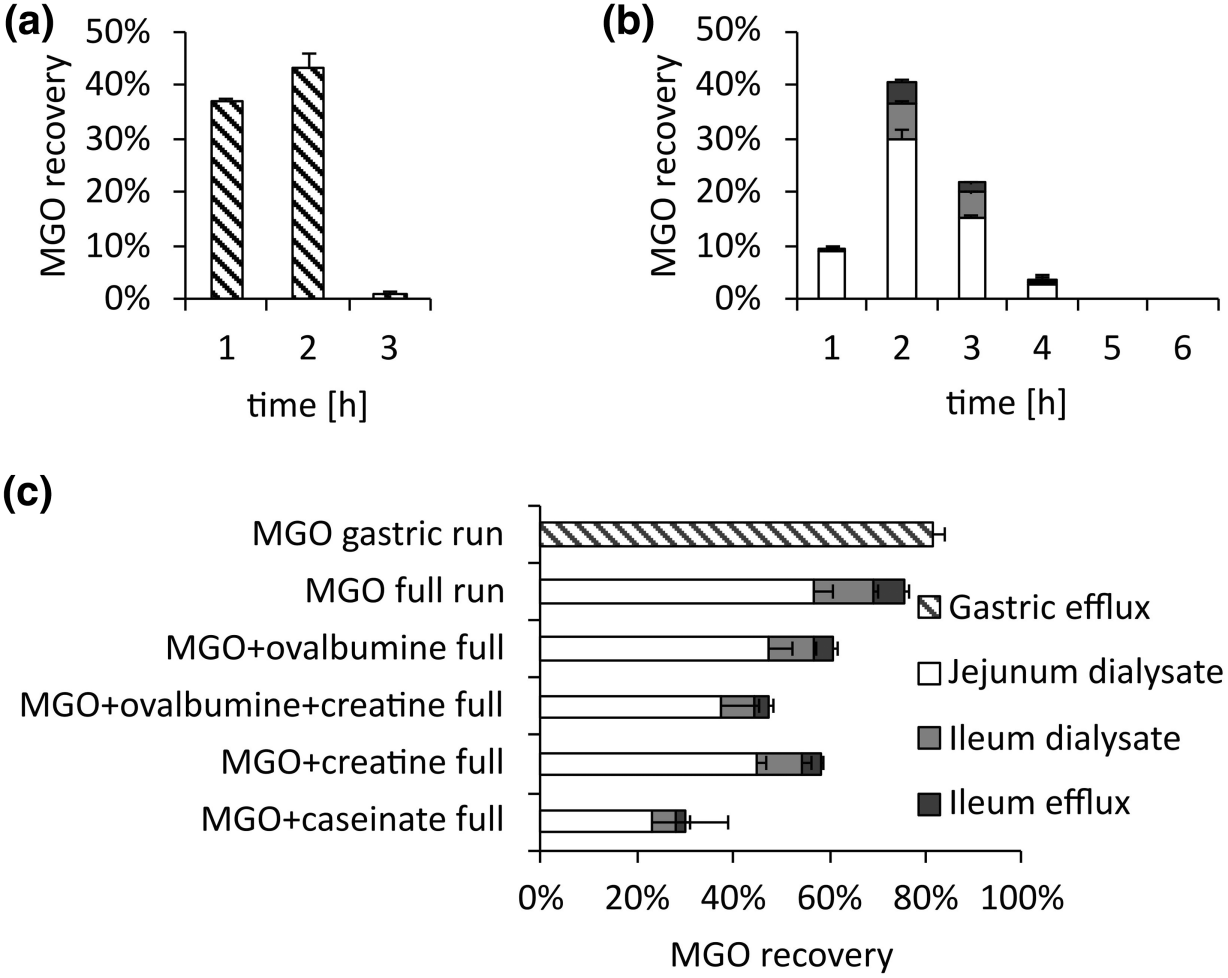
Hourly contents of MGO in the dialysates and efflux of TIM‐1 experiments of MGO without additives in a gastric run (a), in a gastric and small intestinal run without additives (b), and total recovery of MGO with added creatine, ovalbumine, and caseinate (c). Data are presented as mean ± SD, *n* = 2.

After evaluating the applicability of TIM‐1 to study the reactions of MGO during digestion, we now wanted to analyze what happens to MGO during simulated digestion in this model. First, experiments using only the stomach compartment and only MGO as a meal were performed. The samples were taken from the pyloric sphincter (Figure S1, B). As shown in Figure 2a, most of the submitted MGO was found in the samples after one and 2 h. After the run, no MGO was found in the residue. The total recovery of MGO was 82 ± 2%. In static simulated digestion models, the recovery of MGO after a gastric phase without added protein or food matrix is between 95% and 110% (Degen et al., 2013; Treibmann et al., 2022), even in models where a simulated salivary phase was added before the gastric phase (Hamzalıoğlu & Gökmen, 2016). However, in these models, the pH of the gastric stage was immediately set to pH 2. At pH 2, amino groups are protonated, which hinders reactions with MGO. In TIM‐1, the gastric pH is decreased from pH 5.2 to pH 2.0 over 2 h (Figure S2), which corresponds to the conditions in the adult human (Conway et al., 1987). This allows for more reactions between MGO and amino groups of the digestive fluids to take place, which could explain the lower recovery of 82% in TIM‐1 compared to static simulated digestion models.

Gastrointestinal experiments using both the gastric and the intestinal parts of TIM‐1 were also performed. In experiments with only MGO in the meal, 75 ± 4% of MGO was recovered (Figure 2b,c). Most MGO was recovered in jejunum dialysate (57 ± 4%), which is the first dialysis unit, while 12 ± 1% was recovered in ileum dialysate. Only 6 ± 1% of consumed MGO is found in the ileum excretion. This shows that in TIM‐1, MGO is quickly passively absorbed. These recoveries are substantially higher when compared to static simulated digestion models, where 10%–50% of MGO is recovered (Amoroso et al., 2013; Daglia, Ferrari, et al., 2013; Degen et al., 2013; Hamzalıoğlu & Gökmen, 2016; Jiang et al., 2019; Treibmann et al., 2022). Reasons for this might be the quick removal of MGO from the system by the dialysis units mimicking physiological absorption. After the first 2 h, 46% of the initially submitted MGO was already in the jejunum and ileum dialysates. Static systems show that the decrease of MGO during digestion is dependent on time (Degen et al., 2013; Treibmann et al., 2022). Therefore, in TIM‐1, MGO has less time to react with digestive fluids because it is quickly passively absorbed. This entails that more MGO than previously presumed reaches the epithelial cells of the small intestines, where it is available for absorption or reactions with cell components.

Cellular uptake of MGO was proven by submitting MGO to red blood cells and detecting the formation of the MGO‐metabolite d‐lactate (Phillips & Thornalley, 1993). In a study, the absorption of methylglyoxal was analyzed using GIST‐882 cells as gastric cells and Caco‐2 cells as intestinal cells in two different chambers. MGO concentrations decreased after 4 h by 57%–73% (Colombo et al., 2019). However, MGO was solved in RPMI‐1640 medium, which contains amino acids. They could also react with MGO. Since MGO was not analyzed intracellularly, the extent of MGO uptake cannot be extrapolated from this data. In some studies, rat aortic smooth muscle cells (Che et al., 1997) and L6 muscle cells (Riboulet‐Chavey et al., 2006) were incubated with ^14^C_2_‐MGO. These cells incorporated MGO already after 10–15 min, but only 1.8% and 3.1% of MGO in the medium were incorporated into the cells (Che et al., 1997; Riboulet‐Chavey et al., 2006). These works suggest a quick passive uptake of MGO by cells; however, the extent, especially in gastric and intestinal cells, is unclear.

### 3.3 Effect of protein and creatine on the digestion of methylglyoxal

To analyze the reactions between MGO and amino groups further, we added caseinate, ovalbumin, and/or creatine to the meal for TIM‐1. Added protein reduced MGO recovery to 61 ± 6% with ovalbumin and 30 ± 20% with caseinate (Figure 2c). The distribution over time and TIM‐1 fractions was similar in all runs with MGO in the meal (Figure S4). The reduction is in accordance with the literature, where the addition of ovalbumin lowered MGO recoveries from 13% to 7% in static simulated digestion experiments of biscuits (Hamzalıoğlu & Gökmen, 2016). In another study, caseinate reduced the MGO recovery from 19.6% to 13.8% (Degen et al., 2013). Caseinate might decrease the MGO recovery in TIM‐1 more because it precipitates during the gastric phase, which slows the transportation of MGO to the following compartments.

**FIGURE 3:**
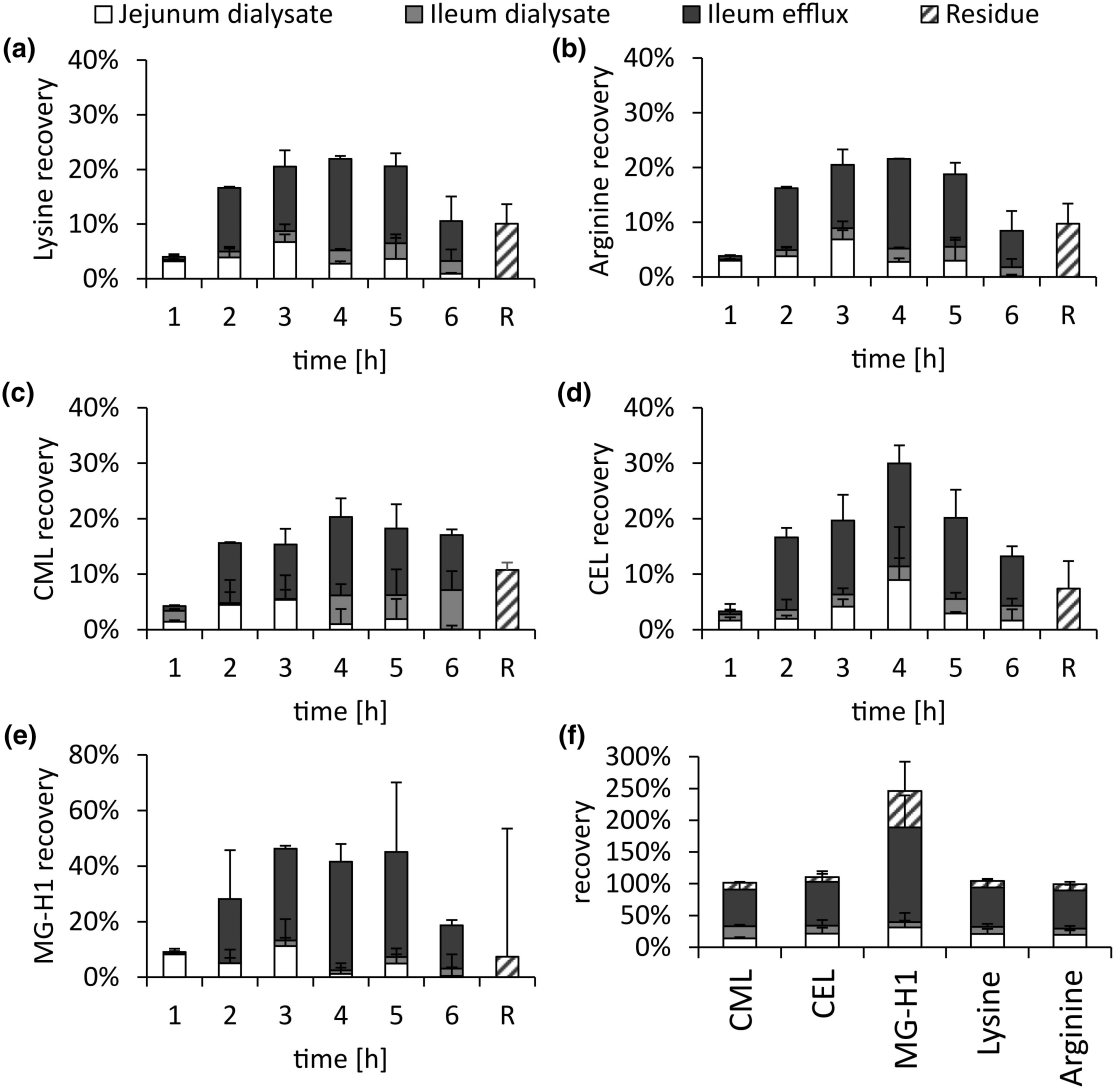
Hourly contents of lysine (a), arginine (b), CML (c), CEL (d), and MG‐H1(e) and total recoveries (f) in the dialysates and efflux of TIM‐1 during a simulated gastrointestinal digestion experiment of ovalbumin. Data are presented as mean ± SD, *n* = 2. R, residue.

Creatine reduced the MGO recovery to 58 ± 1%. In static simulated digestion experiments, only 8% of MGO was recovered in the presence of creatine, while 14% was recovered in the presence of caseinate. Hence, in static simulated digestion experiments, creatine is more potent in reducing MGO concentrations. In other model incubations and simulated digestion experiments, creatine reacted faster with MGO when compared to arginine and lysine (Treibmann et al., 2019, 2022), which is why it was called a ‘scavenger’ for MGO (Löbner et al., 2015). However, in the TIM‐1 experiments, creatine behaves similar to ovalbumin, while caseinate is more potent in reducing MGO concentrations. Reasons for this might be the quick absorption of creatine (Figure 1a and Figure S3a), which reduces the time available for reactions. Protein, on the other hand, must be enzymatically hydrolyzed before free amino acids and peptides can be absorbed. This can be seen as exemplary for lysine (Figure 3a) and arginine (Figure 3b), which both partly remain in TIM‐1 even after 6 h. This leads to a longer duration of amino groups in the system and a longer time to react with MGO. MGO can also be reversibly bound to amino groups. Proteins and non‐absorbable peptides could thereby also deter MGO from being absorbed by forming labile products. The recovery of MGO in TIM‐1 experiments with ovalbumin and creatine added was 47 ± 10%, which suggests an additive effect of ovalbumin and creatine (Figure 2c). This shows that adding amino groups to simulated digestion experiments of MGO leads to decreased MGO recoveries.

When these MGO recoveries are multiplied with its daily intake, which was estimated to range from 0.04 to 0.3 mmol of MGO (Degen et al., 2012; Maasen et al., 2021), it can be assumed that 28–207 μmol/d of MGO reaches intestinal epithelial cells, which might be decreased to 11–84 μmol/d of MGO when simultaneously consumed with creatine or protein. Currently, it is not understood what happens to MGO after it reaches epithelial cells. In a study with 4 participants, no increase in MGO or its metabolite d‐lactate was found in urine after consumption of 500 μmol of MGO (Degen et al., 2013). However, higher habitual intake of dietary MGO was associated with higher plasma concentrations of MGO (Maasen, Eussen, Scheijen, et al., 2022). This suggests the transport of MGO from the gastrointestinal lumen to the plasma. Glyoxalase I was found in intestinal epithelial cells, where it could metabolize MGO before it enters plasma (Baskaran & Balasubramanian, 1987). The cytotoxic effects of MGO on epithelial cells were discussed but seemed to only occur at concentrations higher than what is normally achieved by diet (Amoroso et al., 2013; Daglia, Amoroso, et al., 2013). Transmission cell culture studies with MGO on intestinal cells could help to further understand what happens to dietary MGO.

Also, based on the MGO recoveries in TIM‐1 experiments of the daily MGO intake, 2–18 μmol/d of MGO could reach the colon, which can be decreased to 1–7 μmol/d of MGO when MGO is consumed without protein or other amines. In the colon, MGO could be metabolized to d‐lactate by bacteria like *Escherichia coli* using the glyoxalase pathway (Ferguson et al., 1998) or to acetol and 1,2‐propanedial by bacteria like different *Lactobacillus* strains using thiol‐independent reduction (Gandhi et al., 2018). MGO also has antibacterial effects because it reacts with bacterial protein or DNA and damages the cells (Ferguson et al., 1998; Thierig et al., 2023). Therefore, MGO consumption could affect the microbiota composition in the gut (Brighina et al., 2021).

### 3.4 Formation of methylglyoxal‐derived glycation compounds during digestion in TIM‐1

In the next step, we wanted to investigate possible reaction products of MGO and amines. As shown in Figure 1b,c, 1.3 ± 0.1% of MGO reacted to form MG‐HCr in simulated digestion experiments with creatine, and 0.9 ± 0.4% of MGO reacted to form MG‐HCr in simulated digestion experiments with creatine and ovalbumin (Figure 1c and Figure S3d). Therefore, glycation reactions of MGO take place during digestion.

We also wanted to investigate the MGO‐derived glycation products CEL, which forms from lysine, and MG‐H1, which forms from arginine. CML was analyzed as well, which is the reaction product of lysine and glyoxal and of oxidative fructoselysine cleavage. Fructoselysine is the first stable glycation product of lysine and glucose. The three glycation products CEL, MG‐H1, and CML were also found in the starting material of ovalbumin used for the meal, but not in caseinate. In meals containing ovalbumin, 8 ± 1 μmol of CML, 15 ± 9 μmol of CEL, and 6 ± 1 μmol of MG‐H1 were present. Additionally, the digestive fluids contained some CML, CEL, and MG‐H1. These amounts were quantitated in a digestion experiment with water and subtracted from the amounts measured in the other digestion experiments. During digestion experiments of ovalbumin, CML, CEL. and MG‐H1 were transported through TIM‐1, released from the protein, and absorbed similarly to other amino acids like lysine and arginine (Figure 3). The total recoveries of CML, CEL, lysine, and arginine are around 100%, while the recovery of MG‐H1 is 246 ± 29%. Hence, MG‐H1 was formed during the digestion experiment. This is in accordance with the literature, where the recovery of MG‐H1 was more than 400% in digestion experiments of ginger biscuits in TIM‐1 (van der Lugt et al., 2020). Since no formation of MG‐H1 was observed in digestion experiments of casein, glucose, and lactose, this formation was suggested to stem from glycation intermediates. Other reaction products of MGO and arginine, like *N* ^ε^‐carboxyethylarginine and another hydroimidazolone of MGO and arginine, MG‐H3, can rearrange to form MG‐H1 (Klöpfer et al., 2011). These compounds were not measured here, but they could explain the formation of MG‐H1 during digestion. Around 30% of CML, CEL, MG‐H1, lysine, and arginine were available for absorption, and the recoveries of all compounds increased from the first to the third or fourth hourly fraction and were lower in the final hourly fraction (Figure 3). Therefore, glycation compounds are similarly released into absorbable peptides as unmodified amino acids. Overall, this supports literature, where CML and fructoselysine were released into peptides smaller than 1000 Da during digestion (Hellwig et al., 2014).

**FIGURE 4:**
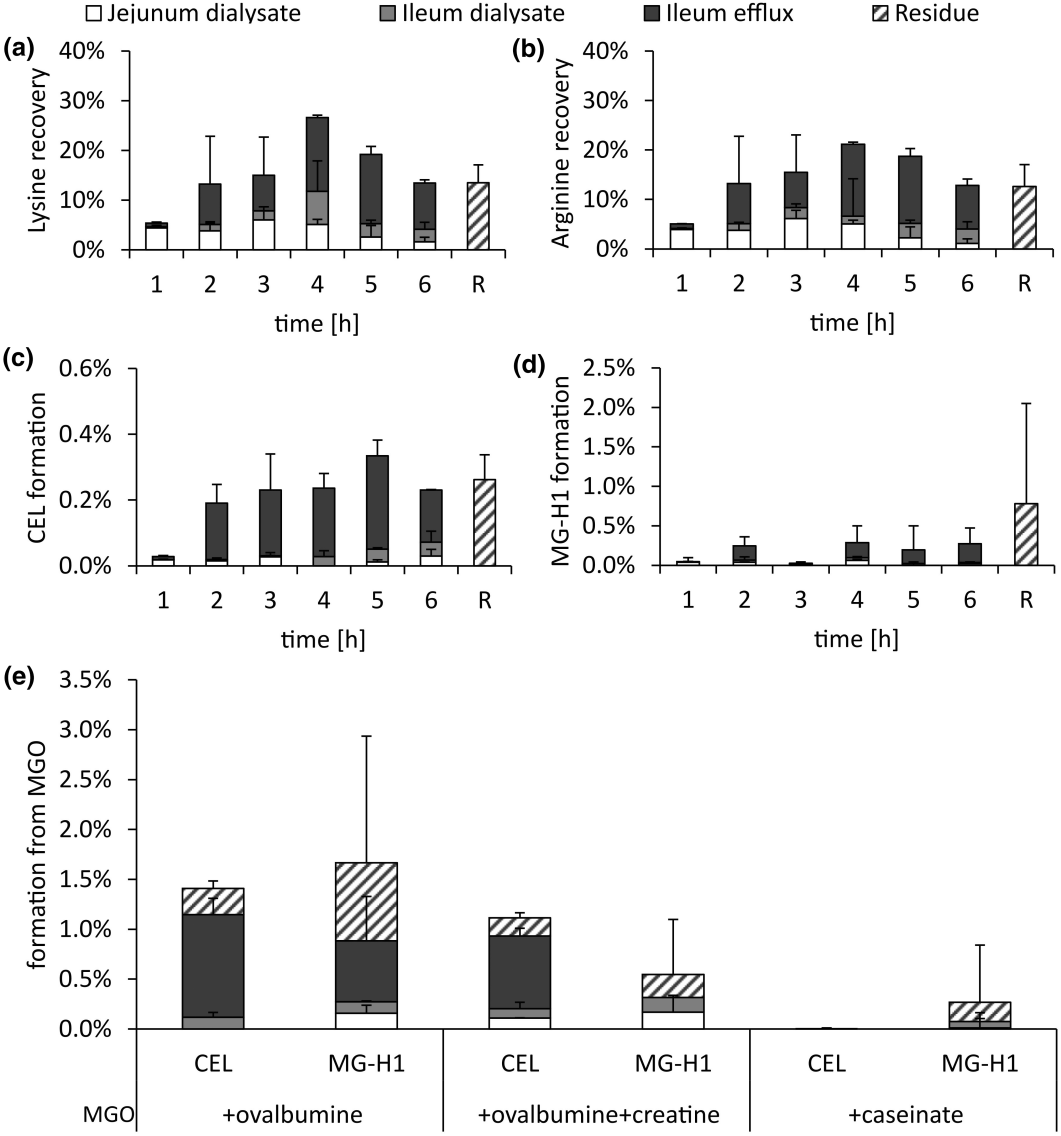
Hourly contents of lysine (a), arginine (b), and formation of CEL (c) and MG‐H1 (d) in the dialysates and efflux of TIM‐1 during simulated gastrointestinal digestion experiments of ovalbumin and MGO, and total formation in experiments of MGO with ovalbumine, ovalbumine and creatine, and caseinate (e). Data are presented as mean ± SD, *n* = 2. R, residue.

To analyze the formation of MGO‐derived glycation products, their amount in the digestion fractions of a simulated digestion experiment of ovalbumin was subtracted from experiments containing MGO and ovalbumin. Adding MGO to digestion experiments of ovalbumin led to a higher concentration of glycated amino acids. As shown in Figure 4c–e, 1.7 ± 1.3% of MGO reacted to form MG‐H1 in ovalbumin, and 1.4 ± 0.3% of MGO reacted to form CEL. In caseinate, the amounts of MG‐H1 and CEL were below the limit of quantitation in many fractions. Therefore, no statement can be made about the formation of glycation compounds during the digestion of MGO and caseinate.

When MGO and ovalbumin were present in the meal, recoveries of lysine and arginine were similarly distributed over time and in the different fractions of TIM‐1 as in experiments without MGO (Figure 4a,b in comparison to Figure 3a,b). Comparably, the addition of MGO or creatine did not significantly affect the amino acid amounts available for absorption in ovalbumin and caseinate for other amino acids as well (Figure S5). Additionally, α‐amino nitrogen was not affected by adding MGO to digestion experiments (Figure S6a). This also implies a similar medium peptide chain length in the TIM‐1 dialysates (Figure S6b). Hence, we could not find evidence that MGO affects the transportation or hydrolysis of proteins during these experiments. The modification of amino acids with MGO after digestion is still relatively low, with approx. 0.2 mol‐%_Lys_ CML, 0.5 mol‐%_Lys_ CEL, and 0.8 mol‐%_Arg_ MG‐H1. In comparison, the digestibility of casein was not affected by mild glycation methods like heating at 60°C for 4 h, resulting in 23 mol‐%_Lys_ fructoselysine. Harsher conditions (60°C for 24 h) resulting in 61 mol‐%_Lys_ fructoselysine, enrichment with CML modification with <4 mol‐%_Lys_, and the cross‐linking amino acid lysinoalanine decreased digestibility (Hellwig et al., 2014). Heating casein with glucose and lactose for 1 h at 100°C also slowed the gastric digestion of caseinate in TIM‐1 experiments (van der Lugt et al., 2020). In simulated digestion experiments of Manuka honey containing high concentrations of MGO, pepsin and pancreatin activities were not affected by reactions with MGO (Daglia, Ferrari, et al., 2013). Therefore, the presence of MGO in relevant food doses does not influence protein digestibility. Hence, the different distribution of glycation compounds in runs with meals containing MGO, in comparison to meals without MGO as observed here, stems from the reaction kinetics of their formation and not from reduced digestibility.

Contrary to the glycation compounds already found in the ovalbumin, CEL and MG‐H1 formed with MGO during digestion were recovered later during the experiments, with the highest amounts in the 5‐h fraction for CEL and in the residue for MG‐H1 (Figure 4c,d). Additionally, only 8% of newly formed CEL and 16% of newly formed MG‐H1 were available for absorption. Therefore, CEL and MG‐H1 are formed later during the run, and less newly formed CEL is available for absorption when compared to CEL already in the meal.

Figure 4e also shows that the addition of creatine to the meal lowered the formation of CEL and MG‐H1 from MGO and ovalbumin. This might be explained by competitive reactions with MGO, which supports the literature stating that creatine is a scavenger for 1,2‐dicarbonyl compounds (Treibmann et al., 2019, 2022). However, the potency of creatine as a scavenger for MGO during digestion is reduced due to its quick absorption (Figure 1a).

To evaluate the daily formation of digestive glycation compounds in vivo, we multiplied the daily intake of 0.04 to 0.3 mmol MGO (Degen et al., 2012; Maasen et al., 2021) with the formation rates of MG‐HCr, MG‐H1, and CEL from MGO in digestion experiments with ovalbumin or creatine and MGO. If all MGO is consumed in combination with protein and creatine, 0.7–5.0 μmol of MG‐H1, 0.6–4.3 μmol of CEL, and 0.5–3.9 μmol of MG‐HCr are formed during digestion. The daily intake with the diet is estimated to be 70–130 μmol for MG‐H1, 7–14 μmol for CEL, and 6.4 μmol for MG‐HCr (Scheijen et al., 2017; Treibmann et al., 2019). Therefore, CEL, MG‐H1, and MG‐HCr formed during digestion from dietary MGO substantially contribute to the total intake of glycated amino acids. However, if MGO is consumed without protein or creatine, e.g. in the form of Manuka honey or coffee, the formation might be significantly lower. These digestive glycation compounds could be absorbed as peptides (Hellwig et al., 2011), released into the blood stream, and added to the free glycation compound pool in vivo.

Since the formation of CEL and MG‐H1 occurs later during digestion, most of these newly digested glycated amino acids will be transported to the colon. Some glycation compounds can be metabolized by the gut microbiome, like CML (Hellwig et al., 2019), pyrraline (Hellwig et al., 2015), and fructoselysine (Bui et al., 2015; Hellwig et al., 2015). Dietary glycation compounds also may affect the gut microbiota composition; however, studies have varying results (Aschner et al., 2023). Glycation compounds formed during digestion should thus be considered in studies researching the effects of dietary glycation compounds and might explain conflicting results in the literature (Nowotny et al., 2018).

## 4 CONCLUDING REMARKS

Overall, we could show that TIM‐1 is a suitable model to study glycation reactions of MGO during digestion by comparing simultaneous digestion of MGO with creatine to the results of a human intervention study. The recovery of MGO as MG‐HCr in TIM‐1 dialysates was 1%, which is close to results from a human intervention study with a 0.2% recovery. In TIM‐1 runs of MGO without added amino compounds, 69% of MGO was passively absorbed and 6% was transported to the colon. With added protein and/or creatine, 28%–57% of MGO were available for absorption and 2%–4% were found in the ileum efflux. These recovery rates are higher when compared to the literature using static simulated digestion models without absorption. Therefore, more dietary MGO may reach intestinal cells than previously presumed. Further studies are needed to investigate how MGO reacts and is metabolized by intestinal cells and the gut microbiome. Simultaneous digestion of MGO with ovalbumin leads to the formation of the MGO‐derived glycated amino acids CEL and MG‐H1. However, based on our results, most of the newly formed MG‐H1 and CEL are not available for absorption and will be transported to the colon. Adding creatine to the digestion inhibited the formation of CEL and MG‐H1. However, the purported MGO‐scavenging potency of creatine is reduced because of its quick absorption.

To demonstrate the relevance of this study, the composition and potential formation of glycation compounds during digestion of a typical German breakfast can be compared. A meal consisting of two bread rolls with jam and sandwich meat, one boiled egg, and one cup of coffee would contain approx. 1.67 mg of MGO (Maasen et al., 2021), 170 mg of creatine (Treibmann et al., 2019), and 27.9 g of protein (nutrition declaration of products from a local supermarket). Additionally, around 0.12 μmol of MG‐HCr (Treibmann et al., 2019), 23.5 μmol of MG‐H1, and 1.7 μmol of CEL (Scheijen et al., 2016) are present in these foods. This meal can therefore be compared to the simultaneous simulated digestion of MGO, ovalbumin, and creatine, and the recovery and formation rates during this experiment are used to calculate the potential distribution of MGO and the formation of glycation compounds. Thus, it can be assumed that from this breakfast, 10.3 μmol of MGO are available for absorption or reactions with epithelial cells, and 0.70 μmol of MGO will be transported to the colon. During digestion, 0.21 μmol of MG‐HCr, 0.09 μmol of MG‐H1, and 0.26 μmol of CEL could form. Hence, glycation compounds formed during digestion contribute to the total consumption of glycation compounds from this typical German breakfast.

In conclusion, glycation compounds are formed during digestion and significantly contribute to dietary glycation compounds, especially to those reaching the colon. Future research should focus on the reactions and metabolization of MGO in intestinal cells and gut microbiota, as well as the physiological effects of glycation compounds formed during digestion.

## AUTHOR CONTRIBUTIONS

**Stephanie Treibmann:** Conceptualization (equal); data curation (lead); formal analysis (lead); funding acquisition (supporting); investigation (equal); methodology (equal); validation (equal); visualization (lead); writing – original draft (lead); writing – review and editing (equal). **Koen Venema:** Conceptualization (equal); investigation (equal); methodology (equal); resources (supporting); validation (supporting); visualization (supporting); writing – review and editing (supporting). **Thomas Henle:** Conceptualization (equal); methodology (supporting); project administration (lead); resources (lead); supervision (lead); writing – review and editing (supporting).

## CONFLICT OF INTEREST STATEMENT

The authors declare no conflict of interest. The funders had no role in the design of the study; in the collection, analyses, or interpretation of data; in the writing of the manuscript; or in the decision to publish the results.

## ETHICS STATEMENT

This study does not involve any human or mammalian testing.

## Supporting information


Data S1.

## Data Availability

The data that support the findings of this study are available on request from the corresponding author.
