# Supplementary material for: Glycation reactions of methylglyoxal during digestion in a dynamic, in vitro model of the upper gastrointestinal tract (TIM‐1)
Source: Food Sci Nutr. 2024 Mar 25;12(7):4702–12. doi: 10.1002/fsn3.4118 (PMC11266905; doi:10.1002/fsn3.4118)
Supplement: Supplementary file 1 — Data S1. [file FSN3-12-4702-s001.docx]

**Glycation Reactions of Methylglyoxal during Digestion in a Dynamic, *in vitro* Model of the Upper Gastrointestinal Tract (TIM-1)**

**Supporting information**

Stephanie Treibmann^1^, Koen Venema^2^, Thomas Henle^1^

1 Chair of Food Chemistry, Technische Universität Dresden, D-01062 Dresden, Germany

2 Centre for Healthy Eating & Food Innovation (HEFI), Maastricht University - campus Venlo, Venlo, The Netherlands

Corresponding author:

T. Henle

Tel.: +49-351-463-34647

Fax: +49-351-463-34138

Email: Thomas.Henle@chemie.tu-dresden.de


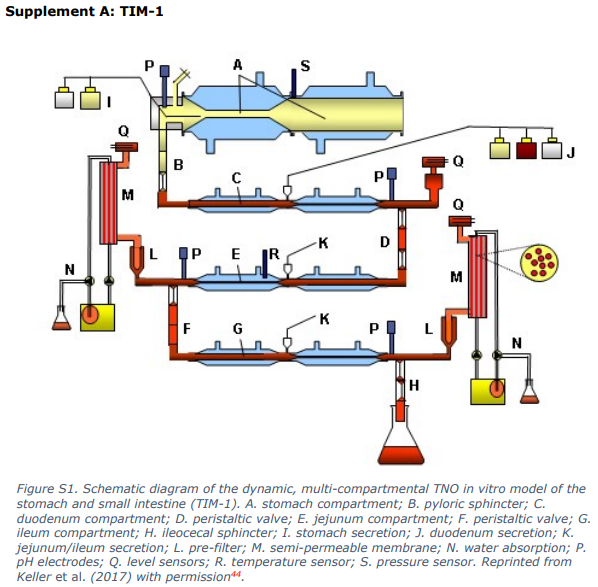


**Figure S1.** Schematic diagram of the dynamic, multi-compartmental TNO in vitro model of the stomach and small intestine (TIM-1). A. stomach compartment; B. pyloric sphincter; C. duodenum compartment; D. peristaltic valve; E. jejunum compartment; F. peristaltic valve; G. ileum compartment; H. ileo-caecal sphincter; I. stomach secretion; J. duodenum secretion; K. jejunum/ileum secretion; L. pre-filter; M. semipermeable membrane; N. water absorption; P. pH electrodes; Q. level sensors; R. temperature sensor; S. pressure sensor. Reprinted from Keller et al. (2017) with permission.

**Figure S2.** Gastric delivery (▲), ileal delivery (●) and the gastric pH (■) curves as mimicked in TIM-1 over time, representing adults.

# Additional Methods

## MG-HCr, creatine and creatinine analysis

After adding 750 μL of solvent mixture (86% of 0.1% formic acid in acetonitrile, 14% of 100 mM ammonium formate in water) and 10 µL of internal standard (5.40 µg/mL D_3_-MG-HCr, 62.5 µg/mL D_3_-creatine, 62.5 µg/mL D_3_-creatinine) to 250 μL of the sample, samples were stored at -18°C for 30 min for protein precipitation, centrifuged (10 min, 4°C, 10600 x g) and subjected to high-pressure liquid chromatography coupled to tandem mass spectrometric (HPLC-MS/MS).

## Acidic Hydrolysis

Briefly, 190 µL of sample was added to 10 µL of internal standard (3.75 µg/mL ^13^C_3_-CEL, 0.75 µg/ml ^13^C_6_-MG-H1, 7.5 µg/ml D_2_-CML) and 0.25 ml of 0.36 M sodium borate buffer (pH 9.5). After that, 0.3 ml of 1 M sodium borohydride in 0.1 M sodium hydroxide was added. Samples were mixed and incubated over night for reduction. Next, 0.9 mL of 11 M hydrochloric acid were added, and samples were incubated at 110°C for 23 h for acidic hydrolysis. After evaporation to dryness, samples were dissolved in 80 μL of 2 M tris(hydroxymethyl)aminomethane buffer (pH 8.2), 260 μL of 0.02 M hydrochloric acid, 100 μL of pure nonafluoropentanoic acid (NFPA) and 1050 μL of distilled water. Samples were subjected to solid phase extraction cartridges (Strata - X, 33 µm, polymeric reversed phase, Phenomenex, Aschaffenburg, Germany), previously consecutively flushed with 2 mL of methanol, 2 mL of methanol/10 mM NFPA (50/50, v/v), and 4 mL of 10 mM NFPA. Cartridges were then washed with 4 mL of 10 mM NFPA and 2 mL of methanol/10 mM NFPA (5/95, v/v) and the analytes were eluted with 2 ml of methanol/10 mM NFPA (50/50, v/v). The eluate was evaporated, solved in 50 µL of solvent mixture (86% of 0.1% formic acid in acetonitrile, 14% of 100 mM ammonium formate in water), and applied to HPLC-MS/MS.

For amino acid analysis, 500 µL of samples were mixed with 500 µL of 6 M hydrochloric acid, incubated at 110°C for 23 h, evaporated to dryness and dissolved in 1 mL of loading buffer. After membrane filtration (0.45 µm), samples were subjected to amino acid analysis.

## Amino Acid Analysis

Amino acids were analyzed on the amino acid analyzer S 433 (Sykam, Fürstenfeldbruck, Germany) using a PEEK column filled with the cation exchange resin LCA K07/Li (150 mm × 4.6 mm, 7 *µ*m, Skykam) and a gradient program. The absorbance of the effluent was recorded with a two-channel photometer simultaneously working at 440 nm and 570 nm, respectively after post-column derivatization with ninhydrin. A commercial amino acid mixture (Sigma-Aldrich, Steinheim, Germany) was used for external calibration and loading and running buffers were purchased from Sykam.

## High-Pressure Liquid Chromatography with UV detection (HPLC-UV) of MGO after Derivatization to 2-Methylquinoxaline

Briefly, samples containing methanol were stored at -18°C for at least 1 hour and centrifuged at 10600 x g and 4°C for 10 min. The supernatant (250 µL) was added to 75 µL of 0.5 M phosphate buffer (pH 6.5) and 75 µL of 0.2 % o‑phenylenediamine. After 16 h incubation in the dark at room temperature, samples were centrifuged and subjected to RP-HPLC with UV detection (312 nm) on a Eurospher 100 RP18 column (250 mm x 4.6 mm, 5 mm particle size with integrated pre-column; Knauer, Berlin, Germany). Acetic acid in water (0.075%) was solvent A and a mixture of 20% solvent A and 80% methanol was solvent B. The starting conditions were 40% solvent B, which stayed at 40% for 1 min and increased linearly to 100% B over 20 min, changed back to 40% B in 4 min, and held there for 7 min at 0.9 mL/min. The separation was performed at 30°C and 20 µL of samples was injected. 2-Methylchinoxaline was used for external calibration.

## High-Pressure Liquid Chromatography with Tandem Mass Spectrometric (HPLC-MS/MS) Detection

An Intrada Amino Acid column (50 × 2 mm, 3 μm, Imtakt (Portland, OR, USA)) was used on a high-pressure gradient system 1200 Series (Agilent Technologies, Böblingen, Germany), consisting of a binary pump, an online degasser, an autosampler, a column oven, and a diode array detector. Separation was performed at 37°C with 0.1% formic acid in acetonitrile as solvent A and 100 mM ammonium formate in water as solvent B. The flow rate was 0.4 mL/min and the injection volume 8 μL. The gradient started at 14% B, stayed there for 3 min, increased to 100% over 7 min, decreased to 14% B within 2 min, and was held at 14% B for 4 min. The system was coupled to a mass spectrometer 6410 Triple Quad (Agilent Technologies) with electrospray ionization in the positive mode, a source temperature of 350 °C, and a capillary voltage of 4000 V. The nebulizing gas nitrogen (nitrogen generator 5183-2003, Agilent Technologies) had a flow rate of 11 L/min and a pressure of 35 psi. Multiple-reaction monitoring mode conditions are listed in the supporting information Table S1. Isotope dilution analysis was used for quantitation.

**Table S1.** Transitions recorded during multiple-reaction monitoring measurement.

|  | Transition | Fragmentor voltage [V] | Collision energy [eV] | Dwell time [ms] | Q/q^†^ |
| --- | --- | --- | --- | --- | --- |
| MG-HCr | 186 → 87 | 105 | 20 | 200 | Q |
|  | 186 → 44 | 105 | 25 | 200 | q |
| D_3_-MG -HCr | 189 → 90 | 110 | 20 | 200 | Q |
|  | 189 → 44 | 110 | 25 | 200 | q |
| Creatine | 132 → 90 | 75 | 10 | 60 | Q |
|  | 132 → 44 | 75 | 20 | 60 | q |
| D_3_-Creatine | 135 → 93 | 90 | 10 | 60 | Q |
|  | 135 → 47 | 90 | 20 | 60 | q |
| Creatinine | 114 → 86 | 105 | 10 | 50 | Q |
|  | 114 → 44 | 105 | 14 | 50 | q |
| D_3_-Creatinine | 117 → 89 | 105 | 10 | 50 | Q |
|  | 117 → 47 | 105 | 14 | 50 | q |
| CML | 205 → 84 | 100 | 20 | 70 | Q |
|  | 205 → 130 | 100 | 10 | 70 | q |
| D_2_-CML | 207 → 130 | 90 | 10 | 70 | Q |
|  | 207 → 84 | 90 | 20 | 70 | Q |
| CEL | 219 → 84 | 100 | 20 | 100 | Q |
|  | 219 → 130 | 100 | 10 | 100 | q |
| ^13^C_3_-CEL | 222 → 84 | 90 | 20 | 70 | Q |
|  | 222 → 130 | 90 | 10 | 70 | q |

|  | Transition | Fragmentor voltage [V] | Collision energy [eV] | Dwell time [ms] | Q/q^†^ |
| --- | --- | --- | --- | --- | --- |
| MG-H1 | 229 → 114 | 75 | 10 | 120 | Q |
|  | 229 → 166 | 75 | 10 | 80 | q |
| ^13^C_6_-MG-H1 | 235 → 171 | 120 | 10 | 120 | q |
|  | 235 → 115 | 120 | 10 | 200 | Q |

† Q, transition used for quantitation; q, transition used for the confirmation of the presence of the analyte.

**Figure S3:** Hourly contents of creatine in the dialysates and efflux of TIM-1 during simulated digestion experiments of creatine (A), creatine and ovalbumine (B) and creatine, methylglyoxal and ovalbumine (C) and formation of MG-HCr in simulated digestion experiments of creatine, methylglyoxal and ovalbumine (D). Total recovery of creatine (E) and formation of creatinine in simulated digestion experiments of creatine with and without added ovalbumine and creatine (F). Data are presented as mean ± SD, n=2.

**Figure S4:** Hourly contents of MGO in the dialysates and efflux of TIM-1 during simulated digestion experiments of MGO with added ovalbumine (A), creatine (B), ovalbumine and creatine (C), and caseinate (F). Data are presented as mean ± SD, n=2. Recovery of MGO alone (without protein or creatine) is shown in Figure 2B.

**Figure S5:** Contents of amino acids in ileum and jejunum dialysates in simulated gastrointestinal digestion experiments of ovalbumin with and without added MGO and/or creatine (A) and of caseinate with added MGO (B) in TIM-1. Data are presented as mean ± SD, n=2. Glx=sum of glutamine and glutamic acid

**Figure S6:** α-amino Nitrogen (A) and medium chain length factor (B) in ileum and jejunum dialysates in simulated gastrointestinal digestion experiments of ovalbumin with and without added MGO and/or creatine, and of caseinate with and without added MGO in TIM-1. Data are presented as mean ± SD, n=2.
